# Utilizing multi-functional neuronal responses during different behaviors to uniquely identify all neurons in the leech ganglion

**DOI:** 10.1186/1471-2202-13-S1-F1

**Published:** 2012-07-16

**Authors:** E Paxon Frady, William B Kristan

**Affiliations:** 1 Department of Neurosciences, UC San Diego, La Jolla, CA 92093, USA; 2 Department of Biological Sciences, UC San Diego, La Jolla, CA, 92093, USA

## 

**Figure 1:**
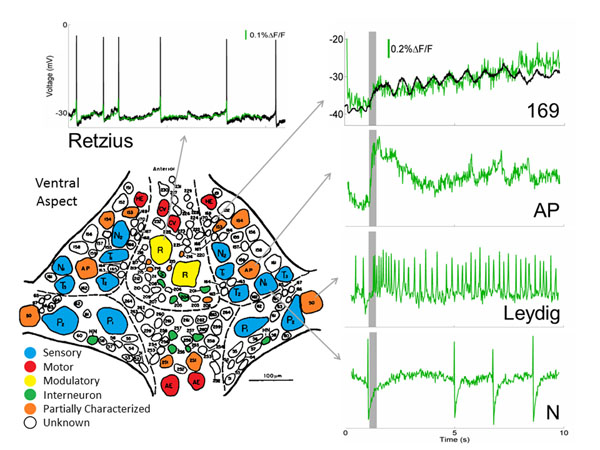
The ventral aspect of the leech ganglion is shown with many of the known cells highlighted in color. About two-thirds are unknown, indicated in white. Several VSD recordings of cells are shown in green. Simultaneous intracellular recordings are shown in black for the Retzius cell and cell 169.

In the leech, we can observe several behaviors – swimming, crawling, shortening, and local-bending – while imaging neuronal activity with voltage-sensitive dyes (VSD) [1]. To understand the underlying neural mechanisms of these behavioral pattern generators, we must understand the functional properties of the neurons and the connectivity between neurons, which requires collecting and synthesizing data across several animals. However, this synthesis is not trivial because more than 70% of the neurons in the leech ganglia have not been identified. We have created a system which utilizes the functional responses of each neuron during these behaviors, as well as during unconventional stimulations, to match homologous pairs of neurons across different animals. Neurons in the leech are almost all multi-functional, in that they are involved in many different behaviors. For a particular neuron, we can use these multi-functional responses as a tag indicating the neuron’s identity. For instance, a neuron in the central packet on the ventral surface can be identified from its neighbors because it oscillates in phase with the dorsal contraction during swimming, and out of phase with the dorsal contraction during crawling. We have developed an algorithm that efficiently combines all of these functional properties as well as each neuron’s morphological properties (soma size and position) to match homologous cells across animals. We have recently developed a new VSD [2] that has given us unprecedented signal-to-noise ratio and temporal resolution of neural activity. This allows us to use cues such as action-potential shape and response timing as further indicators of neuronal identity. Figure 1 shows VSD recordings of several neurons that can now be identified based on their action potentials – such as the Retzius, Leydig, and N cell, or based on response to stimulations – such as the rapid response of the AP cell or the oscillations of cell 169 during swimming. We have used these functional features to identify almost every neuron in ganglion 10 by matching homologs across many different animals. Each animal reveals a different subset of all neurons in the circuit, which are all combined to identify virtually every cell in the leech ganglion. We have used several statistical functional connectivity techniques to estimate the connection profile of these neurons. With these connectivity predictions, we can probe identified cells with micro-electrodes to validate connections and build up the leech connectome with the knowledge of all of the neurons in the circuit and their functional properties during each behavior.
